# Physicochemical Quantitative Analysis of the Oil–Water Interface as Affected by the Mutual Interactions between Pea Protein Isolate and Mono- and Diglycerides

**DOI:** 10.3390/foods13010176

**Published:** 2024-01-04

**Authors:** Ziyan Wang, Jingwen Li, Chao Peng, Bin Li, Qian Shen, Yijie Chen

**Affiliations:** 1 Hubei Key Laboratory for Processing and Transformation of Agricultural Products and College of Food Science and Engineering, Wuhan Polytechnic University, Wuhan 430023, China; wzymelody@163.com; 2 School of Food Science and Nutrition, University of Leeds, Leeds LS2 9JT, UK; 3 National Facility for Protein Science in Shanghai, Shanghai Advanced Research Institute, Chinese Academy of Sciences, Shanghai 201210, China; lijw@sari.ac.cn (J.L.); pengchao@sari.ac.cn (C.P.); 4 College of Food Science and Technology, Huazhong Agricultural University, Wuhan 430070, China; libinfood@mail.hzau.edu.cn

**Keywords:** pea protein isolate, mono- and diglycerides, competition adsorption, quantitative protein proteomics

## Abstract

As a commercially available ingredient, the mono- and diglycerides (MDG) were widely used in a plant protein-based emulsion to provide effective, functional, emulsifying properties. The simultaneous addition of the MDG and pea protein isolate (PPI) was investigated by the methods of interfacial rheology and quantitative protein proteomics. The physicochemical quantitative analysis of the oil–water interface revealed an interfacial stability mechanism for the protein adsorption layer. For a low MDG concentration, the interfacial quantities of vicilin and albumin were increased, which could be attributed to the adsorption rate. For a high MDG concentration, both vicilin and albumin were displaced by MDG and desorbed from the interface, while legumin was more difficult to displace due to its slow adsorption and the complex structure of protein molecules. The protein molecules with the structural rearrangement interacted with MDG, exhibiting potential effects on the interfacial film structure. Combined with some nanotechnologies, the new comprehension of protein-emulsifier interactions may promote food delivery systems. The research aims to develop an in-depth analysis of interfacial proteins, and provide more innovative and tailored functionalities for the application of the plant protein emulsion.

## 1. Introduction

In the food processing industry, many emulsifiers of drink products consisted of proteins and low molecular weight surfactants. This kind of application aims for a long-term stability and satisfactory shelf-life. As one of the typical plant proteins, the pea protein is specifically characterized by its hypoallergenic and nutritional properties. In our previous studies, the pea protein isolate (PPI) showed a prominent emulsifying and foaming ability [1,2]. Considering the interface stability in the long term, synthesized emulsifiers were adopted in the formulas, such as protein and low molecular weight (LMW) surfactants. The mono- and diglycerides of fatty acids commonly named E471 (MDG, in European Union regulation) as food additives were widely applied in foods, such as bread, cream, and drinks [3]. In recent years, the mono- and diglycerides (MDG) were re-evaluated to add in foods for infants and all population groups [4,5]. MDG was one of the nonionic surfactants and dissolved in the oil phase. In the industry, as a commercially available ingredient, MDG was used in petrolatum-based topical emulsions to provide effective, bifunctional, and emulsifying properties [6]. Since the multiple components in food systems, proteins appear with a variety of low molecular weight (LMW) surfactants to stabilize food emulsions or foams. Among these, proteins reacted by forming a viscoelastic adsorbent layer on the surface of the oil droplets, thereby forming a physical barrier to avoid droplet coalescence. As for the physicochemical properties, MDG was applied as an emulsifier due to its polar duality within the molecular structure. The glycerin corresponded to the hydrophilic part, and the aliphatic chains formed the lipophilic counterpart. These properties imparted multiple, effective MDG features for emulsion and foam stabilization [7]. In practical food applications, there are usually mixtures of proteins and LMW surfactants competing for the interfacial region. The LMW surfactants may disrupt the viscoelastic protein adsorbent layer, leading to a reduction in the interfacial stability. In order to avoid the dynamic instability, MDG was added in the formulation step due to its ability to adsorb certain plant-based products at the interface with good efficiency. Therefore, different interface stabilization mechanisms between proteins and LMW surfactants occurred based on complex interface behaviors.

Generally, most low molecular weight (LMW) surfactants could reduce interfacial tension effectively with their rapid adsorption rate [8]. The protein component may form a highly viscoelastic interface network around oil droplets. In theory, the simultaneous addition of PPI and MDG could provide steric hindrance or electrostatic repulsions, preventing coalescence and flocculation between oil droplets, and cooperatively improving the long-term stability of the emulsion [9]. During the process, the protein displacement caused by the LMW surfactants might influence the interfacial stability. Some studies have shown that viscoelastic properties of interfacial films formed by sodium caseinate were affected by the MDG addition, suggesting that the ratio between sodium caseinate and MDG played an essential and critical role in the emulsion [10,11]. Therefore, the displacement of interfacial protein caused by the protein level may consequently change the steric and electrostatic repulsive forces or the conformational properties at the protein-adsorbed layer.

In general, the interfacial properties of protein-surfactant mixed films were investigated by the drop volume tensiometer and multiple microscopy technology [12,13]. It was reported that MDG was added with the phospholipids to promote the stability of the oil–water interface [14]. Generally, these two types of emulsifiers have been widely combined in the food formulas, which could significantly improve the quality and shelf-life of butter [15]. The critical value of the surfactant volume fraction and its associated relaxation time was determined for the emulsion rheological characterization [16]. The formation of the interfacial film was associated with the interactions between film-forming components and the protein displacement by the surfactant. Moreover, this study aimed to investigate the emulsifying stability of PPI-MDG, and the concentration effect of MDG on the interfacial protein by the semi-quantitative method (sodium dodecyl sulfate-polyacrylamide gel electrophoresis, SDS-PAGE). The effect of the MDG addition on the interfacial properties was further analyzed by the quantitative protein proteomics, involving the protein displacement and consequent conformational change.

In this study, we investigated the adsorption behaviors of PPI and MDG to explore the protein quantitative relationships at the oil–water interface. The main point focused on the competitive adsorption of the interface film in order to reveal the stabilization mechanism of food surfactants in applications. Moreover, the nonlinear interfacial rheology was determined to reveal the film interactions. With the stabilizing effects of the PPI-MDG in the interfacial system, the study further provided the theoretical supports for the formulation optimization of plant protein-based dairy products. Moreover, the results were of great significance in the manufacture and consumption development.

## 2. Materials and Methods

### 2.1. Materials

PPI and its major component proteins (vicilin, legumin, and albumin) were prepared from the pea protein powder (Yantai, Shandong, China) according to the previous operations [17,18]. MDG was provided by Aladdin Reagent Company and dissolved in the oil phase. The reagents used for the mass spectrometry experiment were from Sigma–Aldrich Co (Shanghai, China). All other reagents were of an analytical reagent grade (AR grade).

### 2.2. Emulsion Preparation

The pea protein isolate (PPI) was dissolved in deionized water at a concentration range of 0.1–1% (*w*/*v*). Then, MDG was added once in the medium chain triglycerides (MCT) before the stirring process at 50 °C for 10 min. The protein solution and oil phase mixed at 9:1 was pre-emulsified with a high-speed disperser at 8000 r/min for 2 min. Finally, the emulsion was prepared by the high-pressure homogenizer at 40 MPa for 3 cycles (almost 2 min).

### 2.3. Particle Size

The particle size of emulsion droplets was measured by the Malvern Mastersizer 2000 (Worcestershire, UK) with the laser diffraction. Due to the wet dispersion, the refractive indices were 1.45 for the droplets and 1.33 for the dispersant. The volume-weighted mean diameter (D_43_) was obtained through a particle size distribution with three repeated measurements.

### 2.4. The Percentage of Adsorbed Proteins (AP)

The percentage of adsorbed proteins (AP) was determined according to the relevant methods [19]. The emulsion (8 mL) was centrifuged in 15 mL centrifuge tubes (10,000× *g*, 30 min, 4 °C). After centrifugation, the supernatant was collected by the needle filter at 0.45 μm. Then, the protein concentration in the supernatant (*C_S_*) and the initial protein solution (*C_P_*) was measured with the bicinchoninic acid (BCA) protein assay. The percentage of adsorbed proteins was calculated:

(1) $$Percentage\;of\;adsorbed\;proteins\left(\%\right)=\frac{C_{p}-C_{s}}{C_{p}}\times 100$$

### 2.5. SDS-PAGE

The SDS-PAGE profile of interfacial proteins was determined to show the protein subunit changes at different concentrations. After the centrifugation at 10,000× *g* for 30 min, the upper layer of the cream was collected and then dissolved in a phosphate buffer. Protein samples were mixed with the β-mercaptoethanol (β-ME) at 5:1 (*v*/*v*) for an equal concentration, and then subjected to a boiling water bath for 5 min. In addition, the stacking gel and the resolving gel contained 5% and 12% acrylamide, respectively. The constant voltage during the gel electrophoresis was set to 100 V. Finally, the gel was scanned and imaged using a gel imaging analysis system (Universal Hood II, Bio-Rad, Hercules, CA, USA).

### 2.6. Interface Rheological Properties

#### 2.6.1. Interfacial Tension

The interfacial tension at the oil–water interface stabilized by proteins and MDG was determined using an interfacial rheometer (TECLIS, Civrieux-d’Azergues, France). Firstly, the tip of the syringe was immersed into the cuvette containing the protein solution, which were placed on an optical platform between the light source and the high-speed charge-coupled camera [20]. An automated motor device was used to form a droplet at the syringe tip. Meanwhile, the camera was connected to the video screen for a real-time image. The interfacial tension was measured by fitting a model to the droplet shape. Thus, the reduction in the oil–water interfacial tension, caused by adding proteins and MDG, could be analyzed. The whole period was 2–3 h from the adsorption of proteins and MDG to the interfacial stabilization. During the process, the measuring temperature was 25 °C.

#### 2.6.2. Amplitude Sweep

The dilatational viscoelastic modulus was measured using the above method at a 0.1 Hz frequency with a 10% amplitude [21]. After the stable adsorption for 3 h, the linear or nonlinear viscoelastic region of the protein and MDG at the oil–water interface was determined through the amplitude sweep mode. This mode was measured with an amplitude range of 10–30% at a 0.1 Hz frequency. The experiment was performed in triplicate.

#### 2.6.3. Lissajous Plots

According to the related methods, the analysis of Lissajous curves was to reflect the nonlinear rheological response at the interface [22,23]. The oscillation process contained two different changes (expansion and compression) for three active periods and two balanced periods. Therefore, the relationship of interfacial pressure (*π*) versus deformation (*A*) was considered to reveal the properties of interfacial film.

(2) $$∆\pi =\pi -\pi_{0},∆A=\left(A-A_{0}\right)/A_{0}$$

where the *π* and *A* was the droplet’s interfacial pressure and interface area at a certain time point, respectively. Accordingly, the *π*_0_ and *A*_0_ was the initial value.

During the measurement, the interface was compressed and extended in a sinusoidal process. In the extension part of the cycle, the dilatational moduli at the largest extension (*E_LE_*) and the minimum extension (*E_ME_*) were determined, respectively. The S-factor (*S_E_*) during the extension was calculated according to the following formula:

(3) $$S_{E}=\left(E_{LE}-E_{ME}\right)/E_{LE}$$

In addition, in the compression part of the cycle, the dilatational moduli at the largest compression (*E_LC_*) and the minimum compression (*E_MC_*) were used for the calculation of the S-factor (*S_C_*).

(4) $$S_{C}=\left(E_{LC}-E_{MC}\right)/E_{LC}$$

### 2.7. Interfacial Quantitation of Adsorbed Proteins

Regarding the quantitative analysis of the interfacial protein, the most commonly adopted method was the stable isotope dimethyl labeling [1]. The protein samples were firstly digested by trypsin, then labeled by dimethyl (0, +4, +8) quantitation and desalted by a BAKERBOND C18 column. Through these treatments, interfacial protein samples from a 0.5% PPI-prepared emulsion were set as light labeling (0, Light); interfacial protein samples from a 0.5% PPI-0.1% MDG-prepared emulsion were set as intermediate labeling (+4, Intermediate); interfacial protein samples from a 0.5% PPI-0.5% MDG-prepared emulsion were set as heavy labeling (+8, Heavy). Then, samples (L, M, and H) were detected to compare the quantitative difference. The peptide abundance was performed by a mixed quadrupole-TOF LC-MS/MS mass spectrometer (TripleTOF 5600+, AB Sciex, Shimadzu, Kyoto, Japan) equipped with a sodium stream spray ion source, as described in a previous work [1,24]. Thus, the relative abundance of protein was collected from the ratio of the apex signal intensity of survey scan.

### 2.8. Statistical Analysis

The results by independent trials were presented as the means ± standard deviations (SD). All treatments were replicated 3 times unless there was a special statement. An analysis of variance (ANOVA) of experiment data was conducted with SPSS 26.0 Statistics. The statistical differences were set at a significant standard (*p* < 0.05).

## 3. Results and Discussion

### 3.1. The Effect of MDG Addition on the Interfacial Adsorption

**Figure 1:**
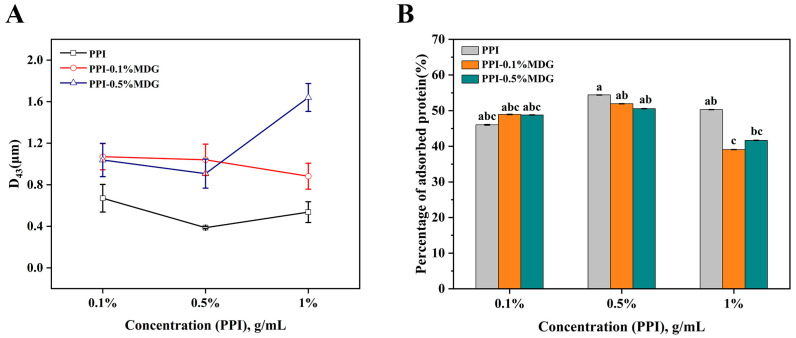
Changes of particle size (D43, **A**) and percentage of adsorbed proteins (AP, **B**) at different concentrations of PPI and MDG. The same lowercase letters marked in the figure represent no significant difference; different lowercase letters indicate significant differences.

Generally, MDG was separated from diglycerides and triglycerides through membrane distillation, and used in bread or margarine for long-term storage. PPI used with MDG was further investigated by an emulsion stability analysis to explore the application of the protein and low molecule weight (LMW) surfactants. As shown in Figure 1, the protein concentration increased from 0.1% to 1%, the droplet size of the emulsion decreased firstly and then increased with the increasing concentration of PPI from 0.1% to 1%. In comparison with the percentage of adsorbed proteins (AP, %), the AP value was the highest at 54.41% when the PPI concentration was 0.5%. After adding MDG, the droplet size was increased. When the PPI concentration was greater than 0.5%, the AP value gradually decreased with the MDG addition. When the protein concentration was 1%, the interface showed the saturation adsorption. This phenomenon was explained by the compact layer after protein adsorption to the interface. The inaccessibility of more protein molecules to the interface prevented the effective adsorption of protein and the intermolecular interactions [25]. According to the principle of LMW emulsifiers, the result was mainly caused by interfacial displacement.

**Figure 2:**
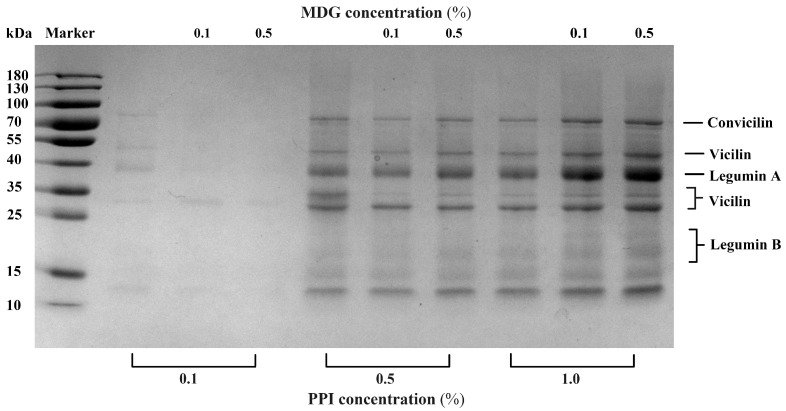
SDS-PAGE profile of adsorbed proteins (Ads.) at the oil–water interface stabilized with PPI and MDG at different concentrations.

The interfacial proteins from the PPI-MDG-stabilized emulsion were collected for the SDS-PAGE measurement, the semi-quantitative method. Through this detection, the differences in the subunit composition of PPI were determined to indicate the protein displacement at the oil–water interface. In Figure 2, the color of protein bands was too light at 0.1% PPI compared with other samples. With the MDG addition, the interfacial protein bands almost disappeared. When the PPI concentration was increased to 0.5%, the SDS-PAGE profiles showed that the interfacial adsorbed proteins (vicilin and legumin B) were decreased according to the missed corresponding bands. The semi-quantitative results suggested that vicilin (25–35 kDa) and legumin (13–20 kDa) were displaced by MDG at the interface. This result was observed by the SDS-PAGE profiles; meanwhile, an accurate measurement confirmed its necessity.

### 3.2. Dilatational Rheological Properties

**Figure 3:**
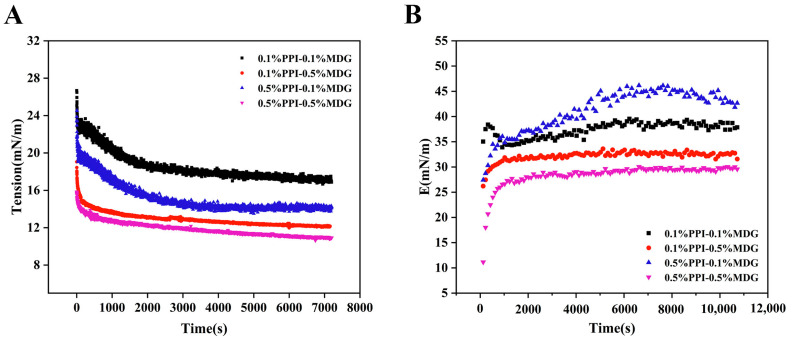
Interface tension (**A**) and interface dilatational modulus (**B**) as a function of time with different concentrations of PPI and MDG at the oil–water interface.

According to the interfacial rheology results in Figure 3, the interfacial tension of samples was decreased at 0.5% MDG. Meanwhile, the interfacial modulus was lower compared with the 0.1% MDG addition. The effect of MDG on the interface tension was more apparent than PPI, which was the typical property of LMW surfactants. The complex modulus decreased significantly with the increase in MDG concentration. In Figure 3B, the complex modulus of 0.5%PPI-0.1%MDG was 43.84 mN/m. Generally, the larger the interfacial complex modulus, the higher the mechanical strength, and this phenomenon was necessary to be further explored [26]. The diffusion rate of 0.1% PPI–0.5% MDG indicated that the protein diffused quickly after the MDG addition. Based on the interfacial kinetic analysis, the interfacial tension decreased at the initial period was attributed to the MDG addition. Therefore, this indicated that proteins with MDG reached interfacial equilibrium more rapidly compared with only protein samples. The faster adsorption and slower rearrangement of the 0.5% PPI–0.1% MDG suggested more adsorbed protein at the oil–water interface and less displacement by the MDG. The result caused less desorption and unfolding of proteins at the interface [27]. At 0.5% PPI, the addition of MDG with less protein rearrangement suggested that the frequency of protein–protein interactions decreased. At the final stage, the interfacial pressure of the 0.5% MDG sample was increased, and the maximum value of the interfacial pressure was 18.78 mN/m for the 0.1% PPI–0.5% MDG. The gradual increase in E with the adsorption time was explained by an intermolecular interaction and protein adsorptions at the interface [28,29]. The result showed that MDG addition played a dominant part in the interfacial tension, and its potential mechanism on the interfacial film was further analyzed from the nonlinear rheology.

**Figure 4:**
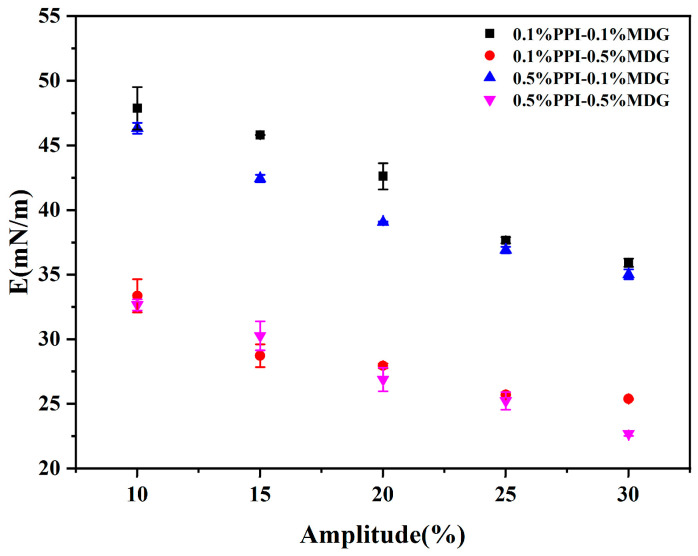
Interface dilatational modulus as a function of amplitude for the oil–water interface stabilized by MDG and PPI at different concentrations. Frequency: 0.1 Hz; deformation amplitude: 10–30%.

According to the relevant research, the properties of an interfacial protein film could define the competitive adsorption behaviors at the oil–water interface [30]. As shown in Figure 4, the complex modulus of the PPI-MDG stabilized interface was determined at an amplitude of 10–30%. The dependence of the interfacial viscoelastic modulus on the interfacial pressure was further estimated. The interfacial complex modulus was decreased with the increasing amplitude, suggesting the nonlinear viscoelastic region with the amplitude range. 

Based on the above analysis, it was determined that the interfacial elasticity of the adsorbed protein film decreased after the protein displacement by MDG. The result indicated that the structural change of the interfacial film was caused by the interface deformation and followed by the interfacial protein rearrangement during the process. It has been reported that the proteins changed their conformation upon adsorption at the oil–water interfaces. This result supported the conclusion.

### 3.3. Lissajous Curves

**Figure 5:**
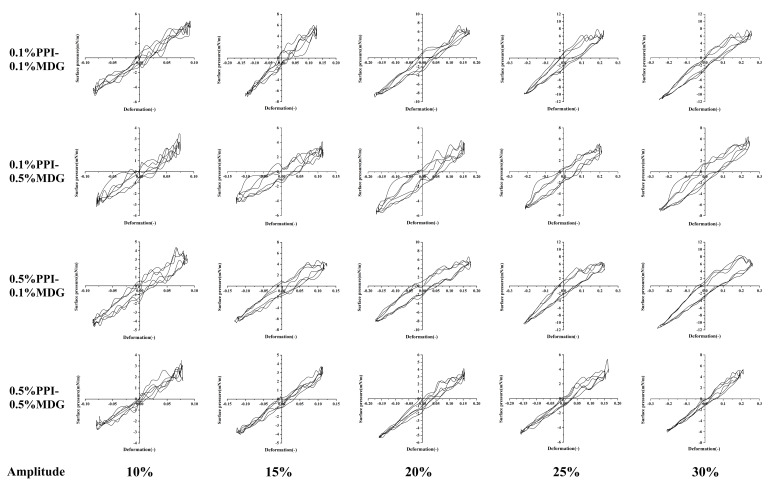
Lissajous plots obtained during amplitude sweeps (10–30%) at the oil–water interface stabilized by PPI and MDG at different concentrations.

In order to further understand the interfacial behavior of PPI-MDG, it was essential to investigate the relationship between the interfacial pressure and interfacial deformation during the amplitude sweep. Therefore, the Lissajous curves were described for the nonlinear interfacial rheological response, and the surface pressure was described as a function of the deformation. According to the previous result, a completely linear shape indicated a purely elastic behavior, while a completely spherical shape indicated a purely viscous behavior of the interface [31,32]. In Figure 5, at lower PPI concentrations, the interfacial proteins were gradually displaced with the increasing MDG. Thus, the interfacial protein film showed a looser distribution, and the compression-hardening response was weakened. At high concentrations of PPI, the interface indicated the dense film structure. In the case of protein displacement, the interfacial film exhibited more significant compression hardening and extension softening. When the film was deformed or disturbed, the protein interactions were weakened to maintain the interfacial stability, or even lead to the interface rupture [33,34].

**Figure 6:**
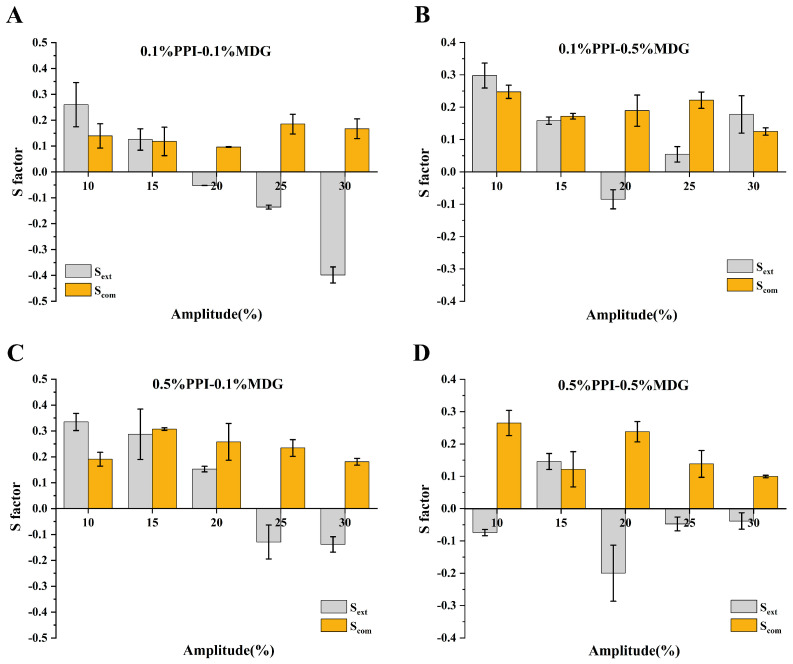
The (**A**−**D**) indicated the different samples. The S factor during extension (S_E_) and compression (S_C_) determined from amplitude sweeps (10–30%) at the oil–water interface stabilized by PPI and MDG.

The lower MDG concentration was used for the faster relaxation of the interfacial tension. The surfactant interfacial properties were related to the interfacial film structure. The interfacial behaviors were attributed to the protein exchanges at the interface, as well as the rearrangement of adsorbed protein at the interfacial film. As shown in Figure 6, the positive value of S factor represented the interface-hardening response, while a negative value of S factor represented the interface-softening response. During the process, all samples showed strain-softening behavior, and the degree of strain softening increased with the increasing amplitude. Similarly, strain-hardening behavior was presented during compression, and the degree of strain hardening increased with the increasing amplitude [35]. Some significant differences in the shape of Lissajous curves were observed at 0.5% PPI. The Lissajous curves narrowed as the MDG concentration gradually increased, suggesting that the interfacial responses were more direct and rapid. The interactions between proteins and LMW surfactants resulted from the hydrophobic or electrostatic interactions, thus affecting the initial structure and interfacial conformation of protein molecules [36,37]. The protein interfacial rearrangement at the oil–water interface was caused by the protein-inherit flexibility and protein hydrophobicity. 

Since LMW surfactants could gradually displace the protein molecules, the addition of MDG might influence the complexation process and the protein composition of the adsorbed layer. At sufficiently high LMW surfactant concentrations (depending on the natural properties of the protein and the small-molecule surfactant), protein molecules could be completely displaced by the LMW surfactants, considering the competitive adsorption mechanism [23]. Combined with the nonlinear rheological behavior, the changes of the interfacial film structure were related with the decrease in interfacial proteins and the weakening of interfacial film strength [38].

### 3.4. Quantitative Analysis of Interfacial Proteins

The interfacial protein composition was determined by mass spectrometry technology (LC-MS/MS) with the method of stable isotope dimethyl labeling. Based on the above experimental results, three sample groups were selected for comparison. In the measurement, the sample of 0.5%PPI was set as the control group, the light standard (L). The sample of 0.5% PPI0.1% MDG was the medium standard (M), and the sample of 0.5% PPI0.5% MDG was the heavy standard (H). The adsorbed protein types and relative contents of three groups at the oil–water interface were detected to further illustrate the relative level of protein displacement by MDG.

**Figure 7:**
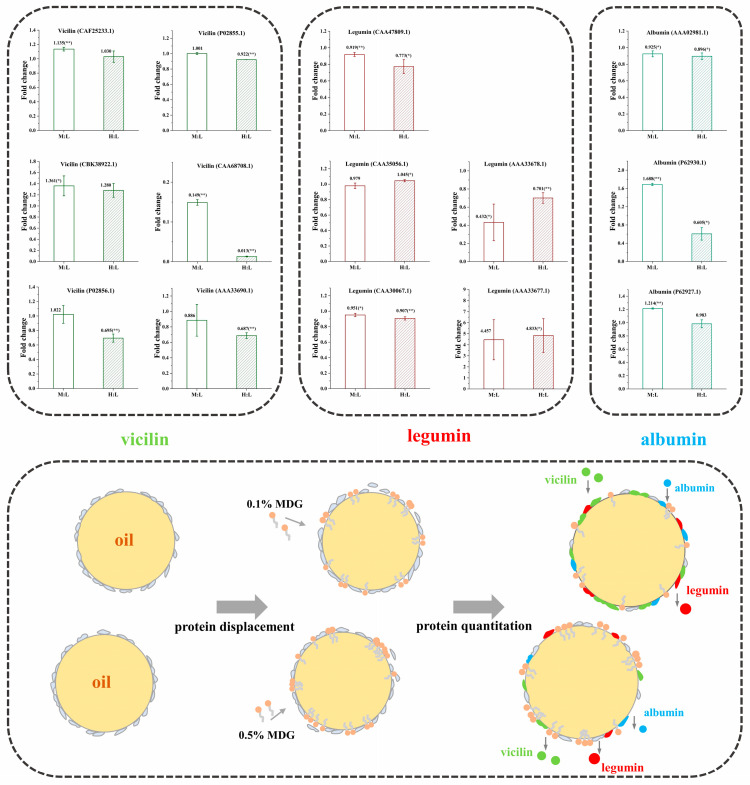
The fold changes of M:L (0.5% PPI–0.1% MDG:0.5% PPI) and H:L (0.5% PPI–0.5% MDG:0.5% PPI) (*, *p* < 0.05; **, *p* < 0.01).

Vicilin (7S) is the trimeric molecule in which each monomer is composed by some subunits. The physicochemical and conformational properties of vicilin depended on its amino acid composition (especially the relative ratio of acidic to basic charged amino acids). Moreover, this ratio influenced its tertiary conformation, quaternary conformation, protein flexibility and thermal stability. In Figure 7, at the quantitative analysis of 0.5% PPI0.5% MDG samples, the vicilin contents were significantly decreased. Combined with the interfacial adsorption results, the increased MDG concentration at 0.5% PPI resulted in the decrease in the interfacial adsorption rate and adsorption capacity. Simultaneously, when the MDG concentration was 0.1%, the vicilin increased. In Table S1, when the MDG concentration was 0.1%, the relative content of vicilin (N4-N5) increased the N17 and N19 as up-regulated proteins. In addition, legumin (11S) is one type of protein with a hexameric structure, in which each monomer contains subunits linked by disulfide bonds. In the 0.5% PPI0.1% MDG samples, the legumin content averagely decreased by 23.27%, indicating that legumins were gradually displaced from the interface. It was speculated that more binding sites appeared at the interface, and other kinds of proteins adsorbed to the interface slowly. There remained a dynamic equilibrium during the process. 

In the comparation of M:L, the content of albumin (2S) increased. When the MDG concentration increased to 0.5%, the 2S decreased by 13.6%, on average. In summary, at a low concentration of MDG, the vicilin and albumin at the interface showed higher content and were preferentially adsorbed at the interface. Moreover, the legumin with the larger molecular weight suggested a slow adsorption rate. At 0.5% MDG, both vicilin and albumin were displaced by MDG and desorbed from the interface, while legumin was more difficult to be displaced due to its slow adsorption and the complex structure of protein molecules [39].

**Figure 8:**
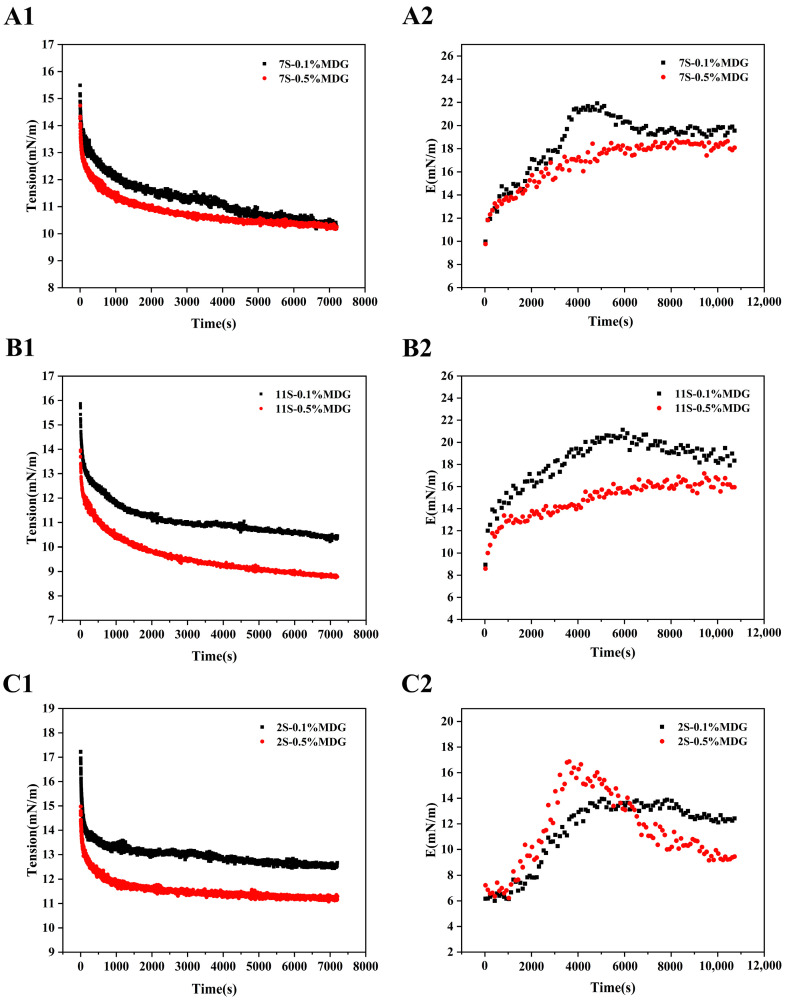
Interfacial tension and dilatational viscoelastic modulus (E) versus time (s) of 7S (**A1**,**A2**), 11S (**B1**,**B2**) and 2S (**C1**,**C2**) at the oil–water interface.

According to the quantitative results, the interfacial rheological behaviors between the component proteins of PPI and MDG were further investigated. The results showed that the interfacial complex modulus increased firstly and then decreased at low concentration of MDG [40]. In Figure 8, the greatest decrease in interfacial tension was observed at 11S when the MDG concentration was 0.5%. The complex moduli of 7S0.5% MDG and 11S0.5% MDG were stable in the range of 16.5–17.5 mN/m, which was higher than that of 2S0.5% MDG. The complex modulus at the interface of the 2S–0.5% MDG samples first increased and then decreased rapidly, suggesting the significant decrease in the content of albumin. In combination with the high concentration of MDG in the quantitative results, the significant desorption of albumin was the explanation for the decrease in the complex modulus at the oil–water interface [41,42].

## 4. Conclusions

During the co-adsorption of PPI and MDG, an interface rheological analysis revealed a significant competitive adsorption. With the increase in MDG concentration, the interfacial proteins were gradually displaced and more loosely distributed, suggesting that the compression-hardening response was weakened. The protein molecules with the structural rearrangement interacted with MDG and showed the potential effects on the interfacial film structure. At low concentrations of MDG, vicilin and albumin were found to be higher at the interface and adsorbed preferentially at the interface. At 0.5% MDG, both vicilin and albumin were displaced by MDG and desorbed from the interface. Meanwhile, legumin with a complex molecular structure was too complicated to displace. Therefore, when LMW surfactants and proteins were used as complex emulsifiers, their synergistic effects at the oil–water interface were the essential factors in selecting the appropriate concentration range.

## Data Availability

The data presented in this study are available on request from the corresponding author. The data are not publicly available due to privacy.

## Supplementary Materials

The following supporting information can be downloaded at: https://www.mdpi.com/article/10.3390/foods13010176/s1, Table S1: Adsorbed proteins identified by LC-MS/MS in the emulsion that was prepared by PPI and MDG at different concentrations and their relative abundance.
