# Supplementary material for: Physicochemical Quantitative Analysis of the Oil–Water Interface as Affected by the Mutual Interactions between Pea Protein Isolate and Mono- and Diglycerides
Source: Foods. 2024 Jan 4;13(1):176. doi: 10.3390/foods13010176 (PMC10779286; doi:10.3390/foods13010176)
Supplement: Supplementary file 1 [file foods-13-00176-s001.zip › foods-2776022-supplementary.pdf]

Table S1 Adsorbed proteins identified by LC-MS/MS in the emulsion that prepared by PPI and MDG at different concentrations and their relative abundance.

N	Accession	Description	MW [kDa]	Fold change (H:L)	Sig.	Up/Down	Fold change (M:L)	Sig.	Up/Down
1	CAA32239.1	vicilin	52.2	1.022 ± 0.039			1.007 ± 0.022		
2	P02856.1	vicilin 14 kDa	14	0.695 ± 0.056	**	↓	1.022 ± 0.123		
3	CBK38917.1	vicilin 47 kDa	49.4	1.035 ± 0.062			1.069 ± 0.037		
4	CBK38922.1	vicilin 47 kDa	49.6	1.280 ± 0.125			1.361 ± 0.179	*	↑
5	CAF25233.1	vicilin, partial	47.3	1.030 ± 0.079			1.135 ± 0.026	**	↑
6	AAA33690.1	vicilin, partial	7.3	0.687 ± 0.037	**	↓	0.886 ± 0.206		
7	CAA68708.1	vicilin precursor, partial	49.5	0.013 ± 0.001	**	↓	0.149 ± 0.007	**	↓
8	P02855.1	provicilin (type A)	31.5	0.922 ± 0.001	**	↓	1.001 ± 0.013		
9	CAB82855.1	convicilin	72	0.952 ± 0.002	**	↓	0.912 ± 0.030	*	↓
10	1713472A	convicilin	71.4	0.806 ± 0.029	**	↓	0.965 ± 0.260		
11	CAP06311.1	convicilin, partial	60.1	0.538 ± 0.044	**	↓	0.869 ± 0.020	**	↓
12	CAP06307.1	convicilin, partial	19.2	0.793 ± 0.084	*	↓	0.750 ± 0.116	*	↓
13	CAA30067.1	legumin	56.9	0.907 ± 0.021	**	↓	0.951 ± 0.022	*	↓
14	CAA35056.1	legumin	59.2	1.045 ± 0.014	*	↑	0.979 ± 0.036		
15	S26688	legumin K	56.2	0.993 ± 0.098			0.897 ± 0.080		
16	CAA47809.1	legumin (minor small)	64.8	0.773 ± 0.084	*	↓	0.919 ± 0.024	**	↓
17	AAA33677.1	legumin precursor, partial	23.4	4.833 ± 1.535	*	↑	4.457 ± 1.822		
18	AAA33678.1	legumin precursor, partial	39	0.701 ± 0.058	**	↓	0.432 ± 0.202	*	↓
19	3KSC_F	prolegumin chain F	56.6	1.025 ± 0.065			0.960 ± 0.046		
20	CAE00466.1	albumin 1	13.9	0.973 ± 0.111			1.043 ± 0.095		
21	P62927.1	albumin-1 B	14	0.983 ± 0.060			1.214 ± 0.009	**	↑
22	P62930.1	albumin-1 E	13.8	0.605 ± 0.137	*	↓	1.688 ± 0.025	**	↑
23	AAA02981.1	albumin 2	26.2	0.896 ± 0.040	*	↓	0.925 ± 0.036	*	↓

Fold change was calculated by ratio of H:L or M:L. The up arrow represented that the fold change was greater than 1, while the down arrow represented that the fold change was less than 1. *, p<0.05; **, p<0.01 (n=3).
